# TELEPROM Psoriasis: Enhancing patient-centered care and health-related quality of life (HRQoL) in moderate-to-severe plaque psoriasis

**DOI:** 10.3389/fmed.2024.1465725

**Published:** 2024-12-10

**Authors:** Gabriel Mercadal-Orfila, Piedad López Sánchez, Aranzazu Pou Alonso, Olatz Ibarra-Barrueta, Emilio Monte-Boquet, Joaquin Borrás Blasco, Nuria Padullés Zamora, Patricia Sanmartin-Fenollera, Cristina Capilla Montes, M. Ángeles Bernabéu Martínez, Salvador Herrera-Pérez

**Affiliations:** ^1^Servicio de Farmacia, Hospital Mateu Orfila, Maó, Spain; ^2^Department of Biochemistry and Molecular Biology, Universitat de les Illes Balears, Palma, Spain; ^3^Servicio de Farmacia, Hospital General de Tomelloso, Tomelloso, Spain; ^4^Servicio de Farmacia, Hospital Universitario de Fuenlabrada, Madrid, Spain; ^5^Servicio de Farmacia, Hospital Universitario de Galdakao - Usansolo, Bizkaia, Spain; ^6^Servicio de Farmacia, Hospital Universitario y Politécnico La Fe, Valencia, Spain; ^7^Servicio de Farmacia, Hospital de Sagunto, Valencia, Spain; ^8^Servicio de Farmacia, Hospital Universitario de Bellvitge, Barcelona, Spain; ^9^Servicio de Farmacia, Hospital Universitario Fundación Alcorcón, Madrid, Spain; ^10^Servicio de Farmacia, Hospital Universitario del Sureste -Madrid, Madrid, Spain; ^11^Servicio de Farmacia, Hospital San Juan-Alicante, Alicante, Spain; ^12^Facultad de Ciencias de la Salud, Universidad Internacional de Valencia, Valencia, Spain

**Keywords:** telepharmacy, psoriasis, patient-reported outcomes measures, quality of life, NAVETA, biological therapy

## Abstract

**Background and purpose:**

Psoriasis is a chronic, immune-mediated inflammatory skin disease that significantly impacts patients’ quality of life. The integration of telepharmacy has the potential to enhance patient care by providing flexible and personalized pharmaceutical follow-up. This study (TELEPROM Psoriasis) evaluates a telepharmacy model for evaluating electronic Patient-Reported outcomes (ePROMs) for individuals with moderate to severe plaque psoriasis in Spain with biological treatment.

**Experimental approach:**

This multicenter prospective quasi-experimental study included 258 adult patients initiating or switching biological/immunomodulatory therapy for moderate to severe plaque psoriasis. Patients were recruited from public hospitals in Spain and monitored through the NAVETA telepharmacy platform over a six-month period. PROMs assessed were the Psoriasis Symptoms and Signs Diary and the Dermatology Life Quality Index at baseline, 1 month, 3 months, and 6 months. Data were analyzed using ANOVA, Student’s *t*-test, multiple regression, and machine learning algorithms to evaluate ePROMs evolution and response and satisfaction with Telepharmacy follow up.

**Key results:**

The analysis revealed significant influences of gender, employment status, educational level, and daily activity, but no effect of age, on responses to Patient-Reported Outcomes questionnaires. Machine learning models, particularly Random Forest (AUC = 0.98) and Support Vector Machine (AUC = 0.96), effectively predicted patient engagement. DLQI scores significantly decreased from 9.33 ± 7.75 at baseline to 4.34 ± 5.86 at 6 months. Similarly, the PSSD - 7 Days questionnaire showed major reductions, with scores dropping from 55.43 ± 29.94 to 30.73 ± 30.66 at 6 months, and 53% of patients reaching a score of 20 or less. Notably, women reported worse scores at all time points compared to men. Regression analysis explained only 13.2% of the variance in PROMs scores, identifying Employment Status and BMI Range as key contributors.

**Conclusion:**

This study demonstrates the efficacy of biologic treatments in significantly improving HRQoL for psoriasis patients. Addressing demographic variables, such as gender, is key for optimizing treatment outcomes and improving ePROMs response rates. Tailored strategies and ML techniques can help identify low-engagement patients and mitigate disparities. Integrating sociodemographic factors into clinical decision-making and patient engagement strategies is fundamental for delivering equitable and comprehensive care.

## Introduction

Psoriasis is a multifactorial immune-mediated chronic and recurrent inflammatory disease of the skin with a disease burden that extends beyond the physical symptoms experienced by patients. The severity of psoriasis is clinically assessed using key measures such as Body Surface Area (BSA) and Psoriasis Area and Severity Index (PASI). BSA quantifies the percentage of the body affected by psoriasis, with moderate psoriasis classified as 3–10% BSA and severe psoriasis as >10% BSA. Moderate to severe psoriasis corresponds to a PASI score > 10, while scores >15 indicate severe psoriasis (1–3). Patients with moderate to severe psoriasis often require systemic therapies or biologics, as topical treatments and phototherapy are typically insufficient to control the disease at this stage. Between 2 and 3% of the world’s population suffers from this disease and 80–90% of psoriasis patients have plaque psoriasis; more than one-third of them present with the moderate–severe form of the disease (4, 5). Biologic and immunomodulatory therapies, such as TNF-*α* inhibitors, IL-12/23 inhibitors, IL-17 inhibitors, IL-23 inhibitors, and PDE4 inhibitors, are essential in managing moderate-to-severe plaque psoriasis due to their targeted mechanisms of action against key inflammatory pathways. These therapies have demonstrated efficacy in achieving significant skin clearance and improving joint symptoms in patients with psoriatic arthritis (6). The impact on the health-related quality of life (HRQoL) for patients with moderate-to-severe psoriasis is key, encompassing various aspects such as physical, emotional, sexual, and economic factors. Demographic characteristics, including gender, age, body mass index (BMI), employment status, education level, tobacco and alcohol consumption, and daily activity levels are known to influence HRQoL and treatment outcomes in chronic conditions like psoriasis (7). Exploring these associations allows for the identification of vulnerable groups and the development of tailored treatment approaches, which are essential to improving overall patient care and outcomes. In addition, the incorporation of patient perspectives by Patient-Reported outcome measures (PROMs) and Patient-Reported experiences measures (PREMs), in treatment decision-making increases patient satisfaction and HRQoL outcomes, which may lead to improved clinical outcomes (8, 9). Importantly, high response rates to these metrics have also been associated with improved HRQoL outcomes, underscoring their value in optimizing patient care (10). However, little is currently known about the impact of sociodemographic variables on response rates to PROMs questionnaires. Electronically administered PROMs (ePROMs) offer several benefits (11), enabling the assessment of a wide range of outcomes, including physical performance, social functioning, psychological well-being, symptom severity, disability, and impairment from the patient’s perspective. ePROMs can be used to support diagnosis, monitor treatment and patient progress, improve communication between patients and healthcare professionals, and facilitate shared decision-making (12). ePROMs have found particular utility in telepharmacy, where they enable remote patient monitoring and personalized care (13, 14).

Telepharmacy makes it possible to guarantee safe and effective pharmaceutical care and also allow for personalized pharmacotherapeutic follow up within much more flexible timeframes, thereby improving the well-being of patients. The implementation of Telepharmacy for drug therapy monitoring is used for management of therapeutic adherence, review of medication, evaluation of clinical outcomes, and ePROMs and ePREMs (15). Telepharmacy, according to the Spanish Society of Hospital Pharmacy (SEFH) document, is the remote provision of pharmaceutical care using information and communication technologies (14).

This study presents a multicenter, prospective, quasi-experimental telepharmacy follow-up study (TELEPROM Psoriasis). The primary aim is to assess the patient-reported effectiveness of initiating or switching biological treatments in adults with moderate-to-severe plaque psoriasis, measured through ePROMs questionnaires. Additionally, the study aims to evaluate how demographic and clinical variables, such as gender, age, and BMI, influence biologic treatment for psoriasis and assess their impact on response rates to ePROMs within this telepharmacy framework. The intervention utilized the NAVETA platform to facilitate asynchronous patient-provider communication and systematically collect ePROMs at defined time points, serving as the primary tool for all follow-up and monitoring (10). We hypothesize that this telepharmacy approach enables consistent collection of ePROMs over time and provides insights into how demographic factors modulate HRQoL outcomes and treatment responses, allowing for a deeper understanding of psoriatic patients’ experiences during biologic therapy. Additionally, this method is also suitable for investigating the impact of sociodemographic variables on ePROMs response rates.

## Methods

### Study design and recruitment

This study is a multicenter, prospective, quasi-experimental pharmacotherapy follow-up conducted within the Spanish public health system. Patients were recruited collaboratively by hospital pharmacists and dermatologists during outpatient pharmacy consultations. Eligible participants were adult patients diagnosed with moderate-to-severe plaque psoriasis initiating or switching biological/immunomodulatory treatment. Moderate psoriasis was defined as 3–10% BSA involvement or a PASI score > 10, while severe psoriasis was characterized by >10% BSA involvement or a PASI score > 15, as assessed by a dermatologist. The follow-up period lasted 6 months, with evaluations conducted at baseline (stage 0), and at 1, 3, and 6 months using validated ePROMs.

### Inclusion and exclusion criteria

#### Inclusion criteria

Adult patients diagnosed with moderate-to-severe plaque psoriasis were eligible if they demonstrated basic digital literacy and access to devices such as smartphones, tablets, or computers to use the NAVETA platform. Patients were required to be initiating or switching treatment with one of the following therapeutic agents: TNF-*α* inhibitors (Adalimumab, etanercept, infliximab), IL-12/23 inhibitors (Ustekinumab), IL-17 inhibitors (Secukinumab, ixekizumab, bimekizumab, brodalumab), IL-23 inhibitors (Guselkumab, tildrakizumab, Risankizumab), or PDE4 inhibitors (Apremilast). These criteria were established based on the project’s focus on evaluating health-related quality of life (HRQoL) outcomes in patients undergoing advanced biologic or immunomodulatory therapies, as determined by the ethical committee and in line with the clinical interests of dermatologists and hospital pharmacists participating in the TELEPROM Psoriasis initiative.

#### Exclusion criteria

Patients under 18 years of age, those unable to provide the required clinical data, or those who did not provide written informed consent were excluded. These criteria ensure that participants are legally and ethically able to consent to the study, and that sufficient and reliable data can be collected to meet the study’s objectives. Additionally, excluding minors aligns with the study’s focus on adult populations receiving biologic or immunomodulatory therapies.

### Study parameters

Baseline demographic, sociological, and clinical data were collected, including sex, age at diagnosis, BMI, education level, employment status, housing, marital status, membership in patient associations, alcohol and tobacco use, regular medications, physical activity level, and the presence of psoriatic arthritis. These comprehensive parameters provided a holistic view of patient profiles and disease burden.

### Evaluation tools

The study used the NAVETA telepharmacy care model, developed in collaboration with FARUPEIB and BiblioPRO, to evaluate treatment impact on patients’ quality of life and disease symptoms. NAVETA operates independently of Electronic Medical Records (EMR), ensuring secure data storage in compliance with Spanish and European data protection standards (10).

The scientific committee of Naveta played a pivotal role in selecting the ePROMs for our study. For psoriasis patients, the chosen PROM standard sets included the Psoriasis Symptoms and Signs Diary (PSSD 7 days) and the Dermatology Life Quality Index (DLQI), both of which are instrumental in capturing the multifaceted impact of psoriasis on patients (16, 17). The PSSD 7 days is a detailed instrument designed to assess the daily symptoms and signs of psoriasis over a 7-day recall period. Patients rated the severity of various symptoms, including itch, skin tightness, burning, stinging, and pain, as well as signs like dryness, cracking, scaling, shedding/flaking, redness, and bleeding, on a scale from 0 to 10. These individual scores were then averaged to derive a comprehensive symptom and sign summary score, ranging from 0 to 100, providing a quantitative measure of the disease’s burden on the patient (18).

In conjunction with the PSSD 7 days, the DLQI was utilized to evaluate the broader implications of psoriasis on the patients’ Health-Related Quality of Life (HRQoL) across six domains: symptoms and feelings, daily activities, leisure, work or school performance, personal relationships, and treatments. The DLQI scores range from 0, indicating no effect on the patient’s life, to 30, signifying an extremely large effect, thus offering a broad perspective on the disease’s impact. Both questionnaires have been adapted to Spanish population (19, 20).

In addition, satisfaction with the telepharmacy program was evaluated using an *ad hoc* Likert scale (0–10), administered at the 6-month follow-up.

Demographic variables were captured using an *ad hoc* questionnaire integrated into the follow-up program. Patients were required to complete this questionnaire before beginning the telepharmacy program, ensuring that all relevant demographic information was systematically collected. Baseline variables included gender, age, body mass index (BMI), employment status, education level, tobacco and alcohol consumption, and daily activity levels. For employment status and education level, patients selected from predefined dropdown options. Employment status included categories such as ‘Employed,’ ‘Unemployed,’ ‘Temporary Medical Leave,’ ‘Permanent Medical Leave,’ and ‘Student.’ Education level options ranged from ‘Primary Education’ to ‘Bachelor’s Degree’ or higher. These variables were chosen for their potential impact on treatment outcomes and their speculative influence on engagement with ePROMs.

### Data collection and administration

**Figure 1:**
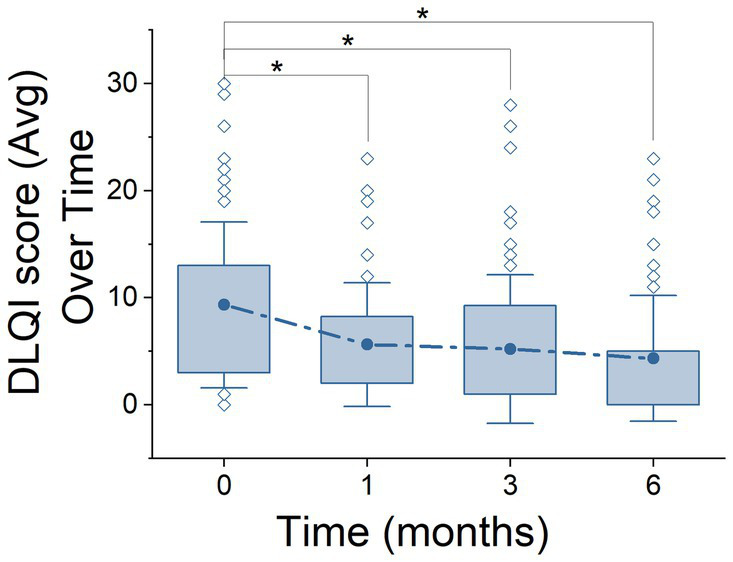
Life Quality Index (DLQI) over the course of treatment stages. Representation of DLQI scores across 6 months using box plots. The plots show the distribution of DLQI scores, highlighting the median, quartiles (25–75%), standard deviation, and outliers at each time point. Solid points above the box plots denote the mean DLQI scores at each stage, providing a direct visualization of the data’s central trend. Asterisks (*) indicate statistically significant differences between stages, according to ANOVA *post hoc* comparison tests.

**Figure 2:**
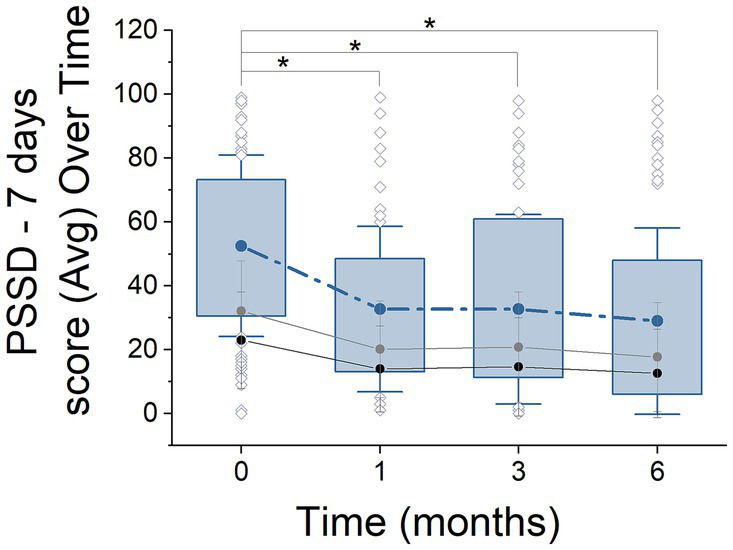
PSSD - 7 days score progression over 6 months of treatment. Box plots illustrate the distribution of PSSD scores (median, quartiles, outliers) at 0, 1, 3, and 6 months. Solid lines represent the mean scores over time, with black and gray lines indicating ‘signs’ and ‘symptoms,’ respectively. Asterisks denote statistically significant changes between time points (ANOVA with Tukey *post hoc* test, *p* < 0.05).

Data were collected at baseline and at 1-, 3-, and 6-month intervals post-treatment initiation. Patients interacted with the NAVETA platform asynchronously, using its integrated chat system for feedback and queries. In this study, we specifically evaluated the number of patients who initiated a conversation through the chat system in relation to the total number of participants in the study, focusing on patient-initiated interactions to assess engagement. The system facilitated real-time data collection and interaction without video or synchronous technologies. Missing data (Specified/Not Classified: NS/NC) management strategies included follow-up reminders, while questionnaire completion rates were monitored at each stage. This longitudinal approach facilitated a dynamic assessment of the treatments’ effectiveness and their impact on both the clinical aspects of psoriasis and the patients’ quality of life, underscoring the value of integrating Patient-Reported outcomes into the comprehensive management and evaluation of dermatological treatments (21). Missing data were handled by excluding patients from specific analyses where relevant data were missing. For instance, if a patient did not report their gender, they were excluded from analyses comparing response rates by gender but remained included in other analyses, such as those evaluating average DLQI or PSSD 7-day scores. This approach ensured that as much data as possible was utilized while maintaining the validity of each specific analysis. We consider this method logical and scientifically robust, aligning with the strategy described by Ranganathan and Hunsberger (22), which supports the selective exclusion of cases for specific analyses to minimize bias and maximize data utility. Outliers identified in the dataset were neither removed nor adjusted during the analysis to preserve the integrity of the data and reflect the natural variability of the population. All analyses, including the box plots presented in Figures 1, 2, display the raw data, including potential outliers.

### Statistical analysis

Data collected were analysed using Origin (Version 2021. Origin Lab Corporation, Northampton, MA, USA.), which provides advanced tools for the analysis and visualization of scientific data. Additionally, Python (Version 3.10) was used to perform complementary statistical analyses and machine learning models, ensuring a robust and comprehensive evaluation of the data. The statistical analyses were conducted as follows:

#### Descriptive and inferential analysis of baseline characteristics and PROM response rates

Baseline demographic and clinical characteristics were summarized using descriptive statistics. Continuous variables were expressed as mean ± standard deviation (SD), and categorical variables were presented as frequencies and percentages. To evaluate the relationship between sociodemographic variables and the response rates to ePROMs, a chi-square analysis was conducted. The dependent variable, “response to ePROMs,” is binary (0 = non-responder, 1 = responder), making it categorical in nature. Given this, an analysis of variance (ANOVA) was not appropriate, as ANOVA assumes a continuous dependent variable. Instead, the chi-square test of independence was selected as it is specifically designed to assess associations between categorical variables (23). The chi-square test was applied to examine the association between response rates and sociodemographic variables such as gender, employment status, education level, daily activity, age range, and BMI range. Additionally, adjusted residuals were calculated to identify which specific categories contributed most to any observed associations. Cramér’s V was used to measure the strength of the associations, offering an effect size for the chi-square test results. Statistical significance was determined using *p*-values derived from the chi-square test, with results reported alongside degrees of freedom (DF) and chi-square statistics (*χ*^2^).

#### Development of a patient classification system: regression and machine learning approaches

To develop a patient classification system, we implemented a 2-step analytical approach. First, we performed multiple regression analyses using Ordinary Least Squares (OLS) and Least Squares techniques to evaluate the relative influence of predictor variables (e.g., response-to-ePROMs (0–1), Stage, Gender, Age Range, BMI Range, Employment Status, Education Level, Smoking, Alcohol Consumption, Daily Activity, Pharmacological Group) on questionnaire response rates. All categorical variables were included simultaneously in the regression models, allowing us to assess their individual contributions to the explained variance (R^2^) without stepwise selection. The percentage contribution of each variable to the total variance was calculated by comparing the residual sum of squares (RSS) of models with and without each variable. Subsequently, machine learning algorithms were employed to predict the likelihood of questionnaire completion and classify patients as “good responders” or “poor responders” based on their engagement with ePROMs. Four algorithms were selected for this purpose: Gradient Boosting, Logistic Regression, Random Forest, and Support Vector Machine (SVM). These models were chosen based on their demonstrated effectiveness in classification tasks and their ability to handle both linear and non-linear relationships (24). Model parameters, such as the number of estimators for Gradient Boosting and Random Forest or the kernel type for SVM, were optimized using grid search and cross-validation techniques to enhance performance (25). A balanced dataset was created using oversampling techniques to address class imbalance, ensuring equitable representation of both responder categories. The dataset was split into 80% for training (*n* = 2,842) and 20% for testing (*n* = 711). Model performance was assessed using the Area under the Curve (AUC) of the Receiver Operating Characteristic (ROC) curve as the primary evaluation metric, along with accuracy, precision, and recall to provide a comprehensive understanding of the classification efficacy. These analyses and model implementations were performed using Python version 3.10 within the Anaconda distribution (current version: 2024.11) (26).

#### Analysis of longitudinal changes in PROMs

To analyze changes over time in ePROMs (PSSD - 7 days and DLQI), repeated-measures ANOVA was employed, with Tukey’s *post-hoc* tests used to identify significant pairwise differences between time points. Levene’s test assessed the homogeneity of variances, ensuring the validity of the ANOVA results. When the assumption of homogeneity was violated, as observed in the NAÏVE group, the Kruskal-Wallis ANOVA (ANOVA K-W) was applied to maintain statistical rigor. These methods ensured a robust evaluation of longitudinal trends in HRQoL outcomes and treatment efficacy across both NAÏVE and SWITCH patient groups. The NAÏVE group consisted of patients who were newly diagnosed and had not previously received any biological systemic treatment for psoriasis. Conversely, the SWITCH group included patients who had previously been treated with at least one biological systemic therapy but were transitioning to a different treatment regimen due to lack of efficacy, tolerability issues, or other clinical considerations. This classification, determined by the hospital pharmacist and recorded in the NAVETA telepharmacy platform. To strengthen the conclusions, power analysis was conducted for the ANOVA, ensuring sufficient statistical sensitivity for key comparisons. To test the potential differential effects on quality of life in relation to treatment, we grouped the patients based on the prescribed drug (see materials and methods). As a result, the groups were Anti-IL-23 Group, Anti-TNF-alpha Group, Anti-IL-17 Group, PDE4 Inhibitor Group, and Anti-IL-12-23 Group. The majority of the study’s patients were receiving treatment with Anti-TNF alpha drugs (59.54%), followed by those treated with Anti-IL-23 (23.18%) and Anti-IL-17 drugs (12.27%). These analyses were performed at a significance threshold of *p* < 0.05. All these statistical analyses and visualizations were carried out using Origin (Version 2021, Origin Lab Corporation, Northampton, MA, USA).

The regression analysis to determine the contribution of sociodemographic variables to PROMs scores was conducted in a similar manner to the approach previously described for predicting PROMs response rates.

Furthermore, we conducted Pearson correlation tests to examine relationships between PROMs questionnaires. Using Chi-square analysis, we assessed the proportion of patients who reached clinically significant improvement thresholds. These analyses were performed using standard statistical software, with a significance level set at *p* < 0.05. Results were expressed as mean ± standard deviation (SD) and analyzed using Origin (Version 2021, Origin Lab Corporation, Northampton, MA, USA).

It is important to note that the observed variations in patient numbers across different stages of the study reflect the natural dynamics of longitudinal data collection in clinical settings. As recruitment was ongoing and patients progressed through the study, they entered different treatment stages at varying times. Consequently, the number of responses analyzed at each timepoint represents those patients who had reached that stage at the time of data collection and completed the corresponding ePROMs. This approach ensures that the analysis accurately reflects the HRQoL outcomes specific to each stage, as supported by prior studies utilizing similar methodologies in telemedicine contexts.

## Results

### Demographic and clinical characteristics of psoriasis patients

Originally, the study enrolled 258 participants, of whom 215 (83%) completed the study with the same biological treatment. The rest of the patients included 8 patients (3%) who voluntarily discontinued their participation, and 2 patients (1%) who could not continue because they lacked the technical skills required to engage in the proposed telepharmacy model. The remaining 34 patients were treated with 2 or more biologicals during the study follow up, given that 11 patients (4%) had to switch due to adverse side effects, and 24 patients (9%) switched because the treatment was not effective. These participants were drawn from various Spanish public hospitals, with the largest contingents from Mateu Orfila General Hospital, Tomelloso General Hospital, and Fuenlabrada University Hospital, which accounted for 18.63, 14.01, and 12.72% of the sample, respectively. Among these participants, 17.3% were diagnosed with concomitant arthritis (see Table 1). Of the 258 patients enrolled in the follow-up program, 37.25% (76 patients) utilized the NAVETA telematic chat system to address treatment-related issues, indicating a significant engagement with digital communication tools. Additionally, the telematic follow-up model received high marks for patient satisfaction, averaging 9.03 ± 1.37 out of 10, suggesting that patients found the digital interaction both effective and satisfactory in managing their treatment and psoriasis issues. This high level of satisfaction underscores the potential benefits and acceptability of integrating telematic solutions in healthcare management combined with in person attendance.

**Table 1:** Characteristics of the population under study.

Characteristic	*n*	%
Hospital		
G.H. Mateu Orfila	41	18.63
G.H of Tomelloso	31	14.09
U.H of Fuenlabrada	28	12.72
U.H of Galdakao - Usansolo	20	9.09
U.H of Bellvitge	20	9.09
U.H Foundation Alcorcón	14	6.36
U.H of the Southeast	13	5.90
U.H San Juan de Alicante	12	5.45
Hospital Son Llàtzer	7	3.18
U.H Infanta Cristina	7	3.18
U.H Vall d’Hebron	7	3.18
U.H Virgen Macarena	6	2.72
G.H of Granollers	5	2.27
General U.H Gregorio Marañón	5	2.27
U.H Germans Trias i Pujol	2	0.90
U.H Son Espases	2	0.90
Gender		
Woman	116	52.72
Man	103	46.81
NS/NC	1	0.45
Employment status		
Employed	129	58.63
Unemployed	34	15.45
Retired	20	9.09
NS/NC	15	6.81
Temporary sick leave	13	5.9
Student	6	2.72
Permanent sick leave	3	1.36
Level of education		
Vocational training	67	30.45
Secondary education	53	24.09
Bachelor’s degree or equivalent	42	19.09
Primary education	36	16.36
NS/NC	15	6.81
Less than primary Education	4	1.81
Doctorate	3	1.36
Smoker		
NS/NC	151	68.63
No	45	20.45
Yes	24	10.90
Alcohol consumption		
Sporadically	96	43.63
Never	60	27.27
Weekends	38	17.27
NS/NC	15	6.81
Daily	11	5
Daily activity		
1-Low >0	135	61.36
0-None	26	11.81
2-Moderate >2	22	10
3-High >4	21	9.54
NS/NC	16	7.27
Age range		
Adulthood (27–59 years)	162	73.63
Elderly (>60 years)	40	18.18
Youth (19–26 years)	16	7.27
BMI range		
N. weight (18.5 ≤ BMI < 25.0)	70	31.81
Obesity (BMI ≥ 30.0)	69	31.36
Overweight (25.0 ≤ BMI < 30.0)	63	28.63
NS/NC	16	7.27
Underweight (BMI < 18.5)	2	0.90
Pharmacological group		
Anti TNF alpha group	131	59.54
Anti IL 23 group	51	23.18
Anti IL 17 group	27	12.27
Phosphodiesterase 4 inhibitor group	8	3.63
Anti IL 12–23 group	3	1.36

This table summarizes the distribution of participants across various hospitals and outlines their employment status. It also provides detailed percentages of participants’ employment types, educational levels, health behaviors (smoking and alcohol consumption), physical activity levels, age ranges, BMI categories, and the pharmacological groups involved in the study. The data underscores the diverse backgrounds of the participants and their varied clinical and demographic characteristics. U.H., University Hospital; G.H., General Hospital; NS/NC, Not Specified/Not Classified.

The distribution of patients was 52.72% female and 46.81% male, with 0.5% preferring not to report their gender. The mean age was 47.29 ± 12.84 years, with the majority in the adult age group (27–59 years) accounting for 73.63% of the cohort. The average Body Mass Index (BMI) was 28.69 ± 6.62, with the majority of patients (31.81%) classified as normal weight (18.5 ≤ BMI < 25.0), followed closely by those with obesity (BMI ≥ 30.0) at 31.3%. Socio-demographic variables such as employment status, education level, tobacco and alcohol consumption, and level of daily activity were analysed. The cohort predominantly consisted of employed individuals (58.63%), with a higher education level (50.1%), 20% tobacco usage, and 43.63% reporting sporadic alcohol consumption. Furthermore, 71.36% of the patients monitored exhibited low to moderate activity levels.

### Analysis of the response profile to patient-reported outcomes questionnaires

For the variable gender, the analysis revealed a significant association with the response rates, as indicated by a Pearson Chi-Square value of 9.38 (*p* = 0.00918). The adjusted residuals highlight that men (Adj. Residual = −2.74) were less likely to respond, whereas women (Adj. Residual = 2.74) were more likely to engage. The analysis of employment status revealed a significant association with patient response patterns to ePROMs, as indicated by a Pearson Chi-Square value (*χ*^2^ = 1238.10, *p* < 0.001) and a substantial Cramer’s *V* value (0.590), highlighting a strong relationship between employment status and response rates. Adjusted residuals show the most pronounced differences between students (residual = 3.3491) and permanent disability leave (−3.3491), with students demonstrating consistently higher response rates. Educational level demonstrated a similarly strong association with ePROMs response rates, with *χ*^2^ = 1238.13 (*p* < 0.001) and Cramer’s *V* = 0.590. Adjusted residuals indicate that individuals with a doctorate or bachelor’s degree had significantly higher response patterns compared to those with primary education or below. Notably, the category of “less than primary education” had a residual of −5.20872, emphasizing lower engagement among less educated participants. Daily activity levels also showed a strong association with response rates, with a *χ*^2^ = 1139.89 (*p* < 0.001) and Cramer’s *V* = 0.566. High activity levels were negatively associated with response rates (adjusted residual = −5.4541), whereas participants with moderate activity demonstrated the highest engagement (residual = 4.75881). BMI range was significantly associated with response rates (*χ*^2^ = 1086.08, *p* < 0.001), with Cramer’s *V* = 0.552 indicating a moderate relationship. Adjusted residuals show that participants with normal BMI (residual = 5.6604) were the most engaged, while those classified as obese (−5.66) demonstrated lower engagement. The relationship between age range and ePROMs completion was not statistically significant, as indicated by a *χ*^2^ = 3.95 (*p* = 0.267) and a low Cramer’s V of 0.03336. Adjusted residuals further confirm minimal variation across age groups, reinforcing that age does not significantly influence response patterns in this cohort (see Table 2).

**Table 2:** Response rates by sociodemographic variables: the table summarizes the chi-square analysis of response rates to PROMs based on collected sociodemographic variables.

Variable	Category	Col% (0)	Col% (1)	Total Col%	Adj, Residual	Cramér’s V	χ^2^	DF	Prob > ChiSq
Gender	Male	53.06	44.89	45.68	−2.74	0.051	9.38	2	0.009
	Female	46.94	54.7	53.95	2.74	0.051			
	NS/NC	0	0.4	0.37	1.18	0.051			
Employment status	Student	0	3.18	2.87	3	0.59	1238.1	6	<0.001
	Permanent Disability Leave	6.99	5.58	5.71	−3.349	1			
	Employed	32.94	62.8	59.92	10.725	1			
	NS/NC	45.48	1	5.29	−34.980	1			
Educational level	Doctorate	0.58	1.74	1.63	2	0.59	1238.13	6	<0.001
	Bachelor’s Degree	6.71	21.31	19.9	6.438	1			
	Less than Primary Education	2.91	1.78	1.89	−5.208	1			
Daily activity level	Low	33.53	65.02	61.98	11	0.566	1139.89	4	<0.001
	High	8.75	9.66	9.57	−5.454	1			
	Moderate	2.92	11.15	10.36	4.758	1			
Age range	Adults (27–59 years)	74.93	74.55	74.58	0.153	0.033	3.95	3	0.267
	Youth (19–26 years)	8.45	6.17	6.39	1.645	0			
BMI range	Normal Weight (18.5 ≤ BMI < 25)	15.16	33.93	32.11	6	0.552	1086.08	4	<0.001

For each variable, the categories display the column percentages of responders (1) and non-responders (0), adjusted residuals highlighting subgroup over- or under-representation, and the strength of association (Cramér’s V). Statistical results, including the chi-square statistic (Chi-square), degrees of freedom (DF), and *p*-values (Prob > ChiSq), indicate significant associations for gender, employment status, education level, daily activity, and BMI. This analysis underscores the influence of sociodemographic factors on engagement with PROMs, informing targeted strategies for improved participation.

**Figure 3:**
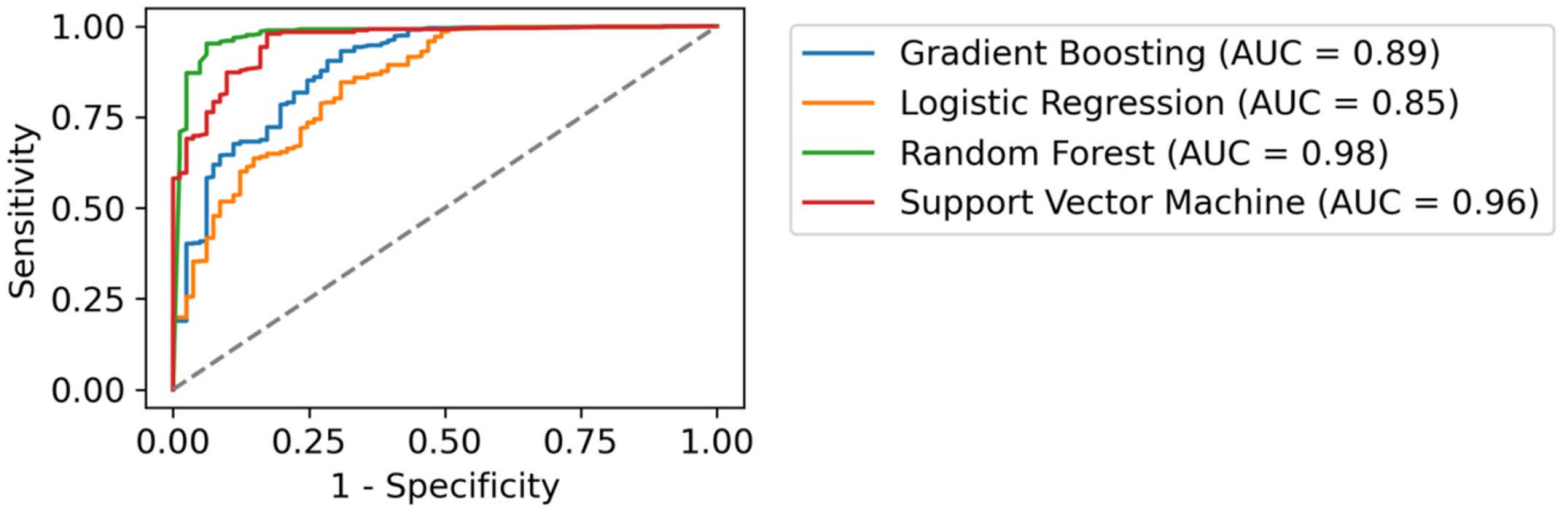
Comparative ROC Curves of Classification Models. This figure illustrates the receiver operating characteristic (ROC) curves for four different machine learning models applied to the same dataset. Each curve represents the trade-off between sensitivity (true positive rate) and 1-specificity (false positive rate) for a distinct classifier. The area under the curve (AUC) values are provided, indicating the performance of each model in distinguishing between the classes. The dashed line represents a random guess.

Subsequently, we conducted multiple regression techniques (OLS, Least Squares) to assess how various predictors (State, Stage, Gender, Age Range, BMI Range, Employment Status, Education Level, Smoker, Alcohol Consumption, Daily Activity, Pharmacological Group) influenced response rates, but these models explained only 2.5% of the variance (Adjusted *R*^2^ = 0.025). Consequently, we shifted to machine learning (ML) algorithms to develop a classification system capable of predicting patient questionnaire completion. After training several models, including Gradient Boosting, Logistic Regression, Random Forest, and Support Vector Machine, both Random Forest (AUC = 0.98) and Support Vector Machine (AUC = 0.96) exhibited strong performance, indicating their efficacy in accurate prediction (see Figure 3).

### Longitudinal assessment of treatment efficacy and patient well-being in dermatology

Next, we focused on analysing the HRQoL and for this aim, we examined the evolution up to 6 months of treatment using two specific metrics designated for psoriasis, the PSSD - 7 days and DLQI questionnaires (Figures 1, 2). For the purposes of this analysis, patients were segregated into two distinct groups: NAÏVE and SWITCH. In addition to analysing these segregated groups, analyses were also conducted on the combined data set, without segregation, to provide a comprehensive overview of the overall treatment effects across all patients.

Regarding the DLQI questionnaire (Figure 1), a total of 250 out of 280 patients (89.3%) completed this questionnaire. A clear decrease was observed from the initial stage from 9.33 ± 7.75 (*n* = 63) to 6 months after treatment (4.34 ± 5.86, *n* = 67), this reduction being significant (ANOVA, *F* = 7.05, *p* = 0.0002, Power = 0.97). In subsequent stages, a significant reduction in DLQI scores was also demonstrated, moving from 5.62 ± 5.76 to 5.20 ± 6.92 over 1 month and 3 months, respectively, (*p* = 0.01, *n* = 62 and *p* = 0.003, *n* = 58 respectively). A similar profile was found when segregating by NAÏVE and SWITCH groups. In the NAÏVE group, a significant difference across stages was detected (Kruskal-Wallis, *χ*^2^ = 20.92, df = 3, *p* = 0.0001), with *post hoc* Dunn’s test showing statistically significant reductions in DLQI scores between the initial stage (0 months) and subsequent stages (1 month: *Z* = 2.87, *p* = 0.024; 3 months: *Z* = 4.09, *p* < 0.0001; and 6 months: *Z* = 3.76, *p* = 0.0002). Levene’s test indicated a violation of homogeneity of variances (*p* < 0.05), necessitating the use of the non-parametric Kruskal-Wallis’s test, as previously described in the Methods section. For the SWITCH group, differences were evaluated using one-way ANOVA (df = 1, *F* = 6.06, *p* = 0.014), highlighting significant improvements in DLQI scores over time (see Tables 3, 4).

**Table 3:** Comparative analysis of the SWITCH group across different treatment stages.

DLQI scores				*Post hoc*			
Group	Stage	Mean	Std	Group 1	Group 2	Mean diff	*p*-adj
SWITCH (*n* = 21)	0	8.569	7.331	0	1	−2.903	0.041*
SWITCH (*n* = 21)	1	5.667	5.419	0	3	−2.857	0.057
SWITCH (*n* = 17)	3	5.712	7.062	0	6	−3.512	0.020*
SWITCH (*n* = 27)	6	5.058	6.542				

This table presents the mean and standard deviation of the Dermatology Life Quality Index (DLQI) scores for the SWITCH group across various stages of treatment (0, 1, 3, 6 months). Pairwise comparisons were conducted using Tukey’s *post hoc* test, with adjusted *p*-values (*p*-adj) provided in the table. Significant differences are marked with an asterisk (*), indicating improvements in DLQI scores between stages.

**Table 4:** Comparative analysis of the NAÏVE group across different treatment stages.

DLQI scores			*Post hoc*			
Group	Stage	Mean	Q1, Q3	Comparison (Groups)	*Z*-value	*p*-adj
NAÏVE (*n* = 42)	0	7.29	1.00, 12.25	0 vs. 1	2.87	0.0243 *
NAÏVE (*n* = 41)	1	4.70	0.50, 7.00	0 vs. 3	4.09	0.00025 *
NAÏVE (*n* = 41)	3	3.81	0.00, 4.00	0 vs. 6	3.76	0.0002 *
NAÏVE (*n* = 40)	6	3.93	0.00, 5.00	1 vs. 3	1.21	0.2247
				1 vs. 6	0.91	0.3613
				3 vs. 6	−0.30	0.7628

This table reports the mean scores and interquartile ranges (Q1, Q3) of the Dermatology Life Quality Index (DLQI) for the NAÏVE group at different treatment stages (0, 1, 3, 6 months). A Kruskal-Wallis’s test was used to assess overall differences across stages, with pairwise comparisons conducted using Dunn’s *post hoc* test. Adjusted *p*-values (*p-adj*) are reported, with significant differences marked by an asterisk (*), indicating reductions in DLQI scores across stages.

No significant differences (*p* < 0.05) were found at the 6-month stage based on gender, tobacco, alcohol consumption or pharmacological group.

**Figure 4:**
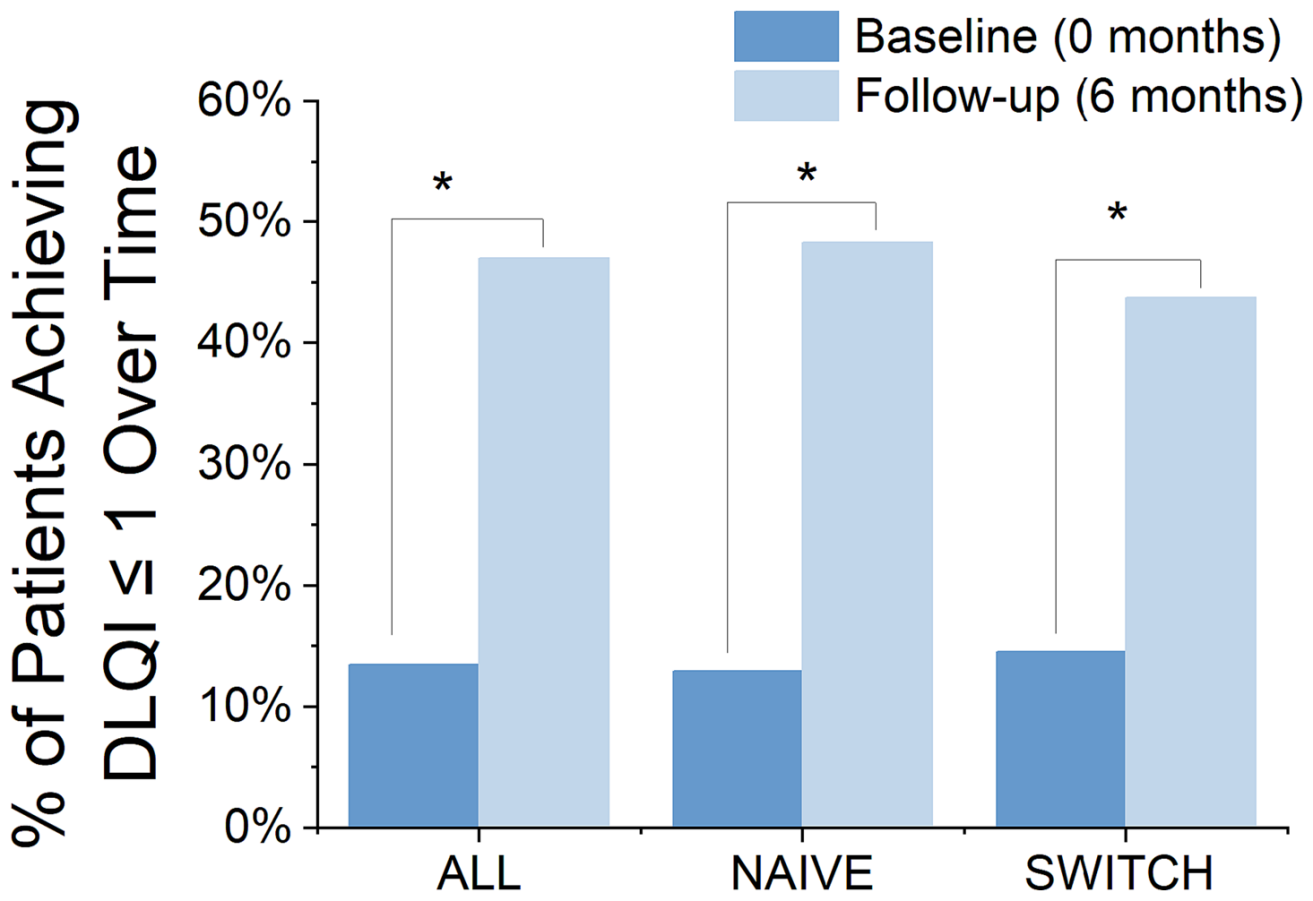
Proportion of Patients Achieving a DLQI Score ≤ 1 at Baseline and After 6 Months, by Group: Comparison of the percentage of patients achieving a DLQI score of 1 or less at baseline and at 6 months, divided into three groups: all patients, NAÏVE patients, and SWITCH patients. Blue bars represent the percentages at baseline, while light blue bars show those at 6 months, demonstrating significant improvements in reported life quality. *Statistically significant difference between baseline and follow-up (chi-square test, *p* < 0.05).

At the 6-month treatment mark, 47% of the patients had achieved a DLQI score of 1 or less, compared to only 13.5% at baseline, indicating a significant change (Chi-square = 22.94, df = 1, *p* = 0.000). A similar profile was observed when analysed by patient groups, with NAÏVE patients showing significant improvement from 12.97 to 48.3% (Chi-square = 17.19, df = 1, *p* = 0.000) and SWITCH patients showing improvement as well from 14.51 to 43.75% (Chi-square = 5.54, df = 1, *p* = 0.01) (Figure 4). Furthermore, among patients who completed the DLQI questionnaire both at the start and at 6 months, 53.48% patients showed a reduction of at least 4 points, which is considered the meaningful change threshold in the DLQI for psoriasis (27). However, when segregating the patients, it was noted that 48.06% of those in the NAÏVE group reached this threshold, compared to 31% of SWITCH patients who achieved a reduction of 4 points in the DLQI at 6 months.

In the PSSD - 7 days questionnaire (Figure 2), 249 out of 280 patients (88.93%) completed the questionnaire. Similarly to the DLQI, a significant reduction in scores was observed, with values decreasing from the initial stage (55.43 ± 29.94, *n* = 65) to the 6-month stage (30.73 ± 30.66, *n* = 68) (ANOVA, *F* = 14.59, *p* = 0.0001, Power = 0.8). This reduction represents an approximate decrease of 44.6%. Significant improvements were also observed in the ‘signs’ and ‘symptoms’ domains, with reductions from baseline to 6 months (*p* = 0.000 and *p* = 0.001, respectively). Additionally, the progression at 1 and 3 months showed consistent decreases across all analyzed domains (ANOVA, Tukey *post hoc* test, *p* = 0.0019, *n* = 58; *p* = 0.0000, *n* = 58, respectively), with reductions of approximately 34 and 40%, respectively.

**Figure 5:**
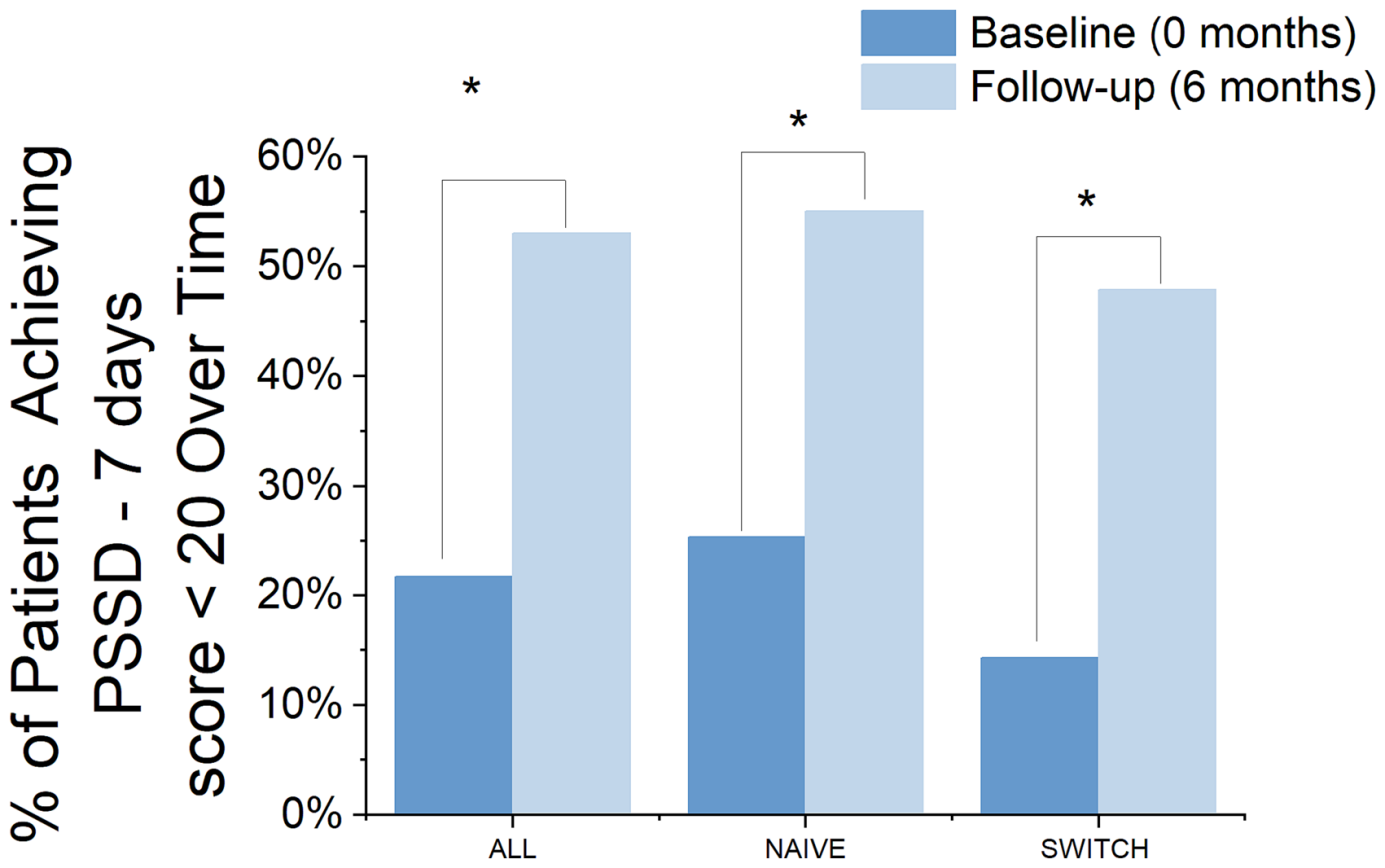
Percentage of Patients Achieving a PSSD - 7 Days Score ≤ 20 Over Time by group. The bar chart displays the percentage of patients achieving a PSSD - 7 days score of 20 or less at baseline and at 6 months, categorized into three groups: ALL, NAIVE, and SWITCH. The comparison between baseline (dark blue bars) and 6 months (light blue bars) highlights significant improvements in achieving the desired outcome. Asterisks denote statistically significant changes between time points.

At the 6-month treatment mark, 53% of the patients had achieved a PSSD - 7 days score of 20 or less, compared to only 21.7% at baseline, marking this change as significant (Chi-square = 14.91, df = 1, *p* = 0.0001). A similar trend was observed in the segregation by patient groups, with NAÏVE patients showing improvement from 25.38 to 55.05% (Chi-square = 8.02, df = 1, *p* = 0.004) and SWITCH patients also showing improvement from 14.02 to 47.91% (Chi-square = 7.04, df = 1, *p* = 0.007) (Figure 5).

The analysis of the variables modulating response at the 6-month stage, we found that men achieved a lower score (ANOVA, *F* = 7.96, df = 1, *p* = 0.0062, Power = 0.8) of 20.14 ± 25.85 compared to women, who reached a score of 39.88 ± 31.52. This indicates that gender plays a significant role in modulating the response to treatment or conditions being measured by the PSSD - 7 days at this stage. Conversely, other variables such as age, educational level, or tobacco and alcohol consumption showed no significant effect (*p* > 0.05) on the PSSD 7-day scores.

Additionally, among the patients who completed the PSSD - 7 days questionnaire both at the start and at 6 months of the study, significant reductions in scores were observed. Specifically, 43.07% patients had reduced their score by at least 15 points, 10% patients had reduced their score by at least 25 points, and 29.23% patients had reduced their score by at least 30 points. When analyzing the percentages of reduction relative to patient type, among NAÏVE patients, 30% reduced their score by 15 points, 10.76% by 25 points, and 36.15% by 30 points. In contrast, in the SWITCH group, the reductions across these thresholds were 16.15, 3.84, and 20%, respectively. These results provide evidence that patients have experienced clinically significant improvements according to the thresholds established in reference (28). However, as demonstrated, a higher proportion of NAÏVE patients achieved these significant improvement thresholds.

Importantly, we demonstrate that during the follow-up period, a moderate yet significant Pearson correlation was observed between the two ePROMs across all stages: Stage 1 (95% CI = 0.17–0.60; *p* = 0.0012), Stage 3 (95% CI = 0.29–0.68; *p* = 0.0000), and Stage 6 (95% CI = 0.01–0.46; *p* = 0.0421).

Finally, to understand how sociodemographic variables may influence the scores of both PROMs, a multiple regression analysis (OLS, Least Squares) was conducted to evaluate the relative contribution of various predictors on the outcomes of both ePROMs. For the PSSD - 7 days, the model revealed that it could explain up to 13.2% (*R*^2^ = 0.132) of the variability in the data. Although this percentage of explained variance by the model is relatively small, it is significant (*F* = 5,252, *p* = 0.000). Upon exploring the individual contributions of the variables, Employment Status (5.47%) and BMI Range (5.05%) were found to be the most influential factors. Conversely, the model exhibited slightly lower explanatory power (*R*^2^ = 0.09, *F* = 3,998, *p* = 0.000) in the analysis of the DLQI questionnaire, where Employment Status (7.97%) and Education Level (2.04%) emerged as the variables with the greatest weight. However, caution is advised in interpreting these results, given that the level of adjusted *R*^2^ in both cases does not reach the desirable threshold (29).

## Discussion

Psoriasis is a chronic systemic inflammatory disease with multi-organ involvement, frequent comorbidities like arthritis, and significant psychological, economic, and social burdens (30). A comprehensive approach to psoriasis care should include ePROMs to evaluate HRQoL, symptom severity, and treatment impact. Engaging patients as active participants through ePROMs supports personalized care, improves decision-making, and enhances adherence and satisfaction. Our study, led by hospital pharmacists in collaboration with dermatologists, utilized routine ePROMs collection to monitor treatment effectiveness, identify issues, and positively impact patient management and satisfaction in real-world settings (31, 32).

### The main findings are as follows

#### Response modulation by demographic and clinical variables

Most existing studies primarily examine sociodemographic variables in relation to treatment efficacy (33–36), such as the work by Warren et al. (35), which examined demographic, social, and clinical predictors of biologic therapy effectiveness in psoriasis, or Scala et al. (36), which explored how demographic and socioeconomic characteristics influence therapeutic decision-making. However, to date, we are unaware of research examining the relationship between sociodemographic variables and response rates to ePROMs in psoriasis. Our findings contribute to addressing this gap by highlighting the significant influence of variables such as gender, employment status, and BMI not only on the interpretation of treatment efficacy from an HRQoL perspective but also on ePROMs data collection itself. For instance, gender differences may impact response behavior, potentially reflecting underlying variations in health-seeking tendencies, perception of disease burden, or engagement with telepharmacy tools. Addressing these disparities through tailored strategies could enhance male participation in ePROMs data collection. Similarly, participants with moderate levels of physical activity demonstrated the highest engagement, suggesting that individuals engaging in more intense or frequent physical activity may perceive themselves as healthier and, as a result, may be less inclined to respond to ePROMs. Meanwhile, the relationship between age range and ePROMs completion was, surprisingly, not statistically significant. To better understand these factors, we used ML algorithms, including Random Forest and Support Vector Machine, to develop a more precise classification system. Both algorithms demonstrated strong performance, with high AUC scores (Figure 3), thus integrating ML techniques to classify patients with varying ePORMs response rates. This approach improves segmentation and helps professionals identify patients at higher risk of low engagement. This aspect has important implications for treatment evaluation, as higher response rates to PROMs have been consistently associated with improved HRQoL outcomes (37).

Therefore, while the existing literature rarely explores the impact of demographic factors on response rates to psoriasis-specific ePROMs, our findings underscore the potential for targeted reminders and tailored communication strategies to mitigate disparities. This emphasizes the importance of addressing barriers to participation in PROMs, particularly among subgroups with lower response rates, as these groups may experience a reduction in their HRQoL due to insufficient adherence to follow-up programs based on ePROMs, such as our TELEPROM Psoriasis initiative.

#### Quality of life outcomes

Our study observed a notable reduction in DLQI and PSSD - 7 days scores over time, indicating significant improvement in HRQoL for patients on biologic therapies. These observations align with previous research documenting enhanced quality of life in psoriasis patients undergoing biologic treatments, which appear more effective than non-biologic therapies in this regard (38–41). This study contributes to the existing evidence by comprehensively evaluating DLQI and PSSD - 7 days scores across multiple time points, highlighting the sustained impact of biologic therapies on HRQoL. Furthermore, significant improvements were noted in both NAÏVE and SWITCH groups, with biologic-naïve patients typically showing a stronger response, consistent with studies indicating that biologic-naïve patients tend to respond better to treatment than those who have previously received biologics (42).

Additionally, our results align with previous findings while providing valuable insights into the influence of demographic and clinical variables, particularly gender, on treatment responses, paving the way for more tailored strategies. Gender differences in PSSD - 7 days scores suggest that women may report higher symptom severity or experience a greater impact from their condition, potentially due to biological factors, differing pain perceptions, or socio-cultural influences on symptom reporting. These findings support the need for gender-specific adjustments in biologic-treatment approaches and highlight the importance of incorporating gender as a key variable in future research. Additionally, the regression analysis underscores the limited explanatory power of demographic and clinical variables on ePROMs scores, with employment status and BMI range significantly affecting PSSD - 7 days outcomes, while employment status and education level influence DLQI scores. The low adjusted *R*^2^ values, however, indicate that other unmeasured factors likely contribute to these outcomes, underscoring the need for further investigation. Nevertheless, it is evident that sociodemographic variables have a clear impact even among psoriasis patients receiving biologic treatments. What remains uncertain, however, is the relative weight of these variables compared to others, such as clinical history or treatment-specific factors, warranting a more comprehensive future analysis.

Additionally, as shown in previous studies (43–45), we found that quality of life metrics PSSD - 7 days and DLQI are correlated; however, our study uniquely demonstrates that this correlation remains consistent across all stages of treatment in a Spanish population. This sustained correlation highlights the stability of patient-reported experiences over time and underscores the continued impact of treatment interventions. Furthermore, it provides clear evidence of the suitability of these two ePROMs for monitoring patients with psoriasis.

One limitation of this study is the absence of a control group that did not receive follow-up via the NAVETA Telepharmacy platform. This lack prevents a direct comparison between standard care and telematic follow-up, potentially obscuring the specific contributions of telepharmacy to patient outcomes. Additionally, while the DLQI and PSSD - 7 days questionnaires were selected to assess HRQoL in this study, alternative questionnaires could provide different insights. The choice of these specific tools, as opposed to others available in the field, may influence the perceived scope and nature of the findings. Furthermore, the recruitment of patients was not uniform across all participating centers, which might lead to variability in the data that could affect the generalizability of the study results. These factors collectively underscore the importance of cautious interpretation of the study outcomes and suggest areas for methodological enhancement in future research.

## Conclusion

Our study highlights the significant role of dual (remote and in-person) assistance in combination with ePROMs programs in psoriasis, as an embodiment of value-based care that centers around the patient as the fundamental axis of our pharmacological and clinical interventions. Patients report high satisfaction marks for this dual clinical follow up satisfaction. This approach not only supports shared decision-making but also boosts patient empowerment, enabling them to take an active role in their healthcare management. These initiatives are essentials for optimizing treatments and improving health outcomes, aligning with global efforts to manage healthcare costs effectively without compromising care quality.

In conclusion, we have shown that sociodemographic variables play a critical role not only in evaluating the impact of biologic treatments on HRQoL in psoriasis but also in improving response rates to follow-up programs based on ePROMs. In this sense, ML techniques can support the identification of patients at risk of low engagement and optimize strategies to address disparities. Biologic therapies have demonstrated significant efficacy in reducing the burden of psoriasis on HRQoL, particularly among men and biologic-naïve patients. Furthermore, the strong and consistent correlation between DLQI and PSSD - 7 days scores underscore their suitability for inclusion in standard sets for long-term monitoring. These findings highlight the value of integrating these metrics into telemedicine initiatives, such as TELEPROM Psoriasis, to ensure more comprehensive and tailored patient care.

## Data Availability

The raw data supporting the conclusions of this article will be made available by the authors, without undue reservation.
